# Acute Ischemic Stroke in COVID-19: A Case-Based Systematic Review

**DOI:** 10.3389/fneur.2020.01031

**Published:** 2020-09-25

**Authors:** Tissa Wijeratne, Carmela Sales, Leila Karimi, Sheila Gillard Crewther

**Affiliations:** ^1^Neurology & Stroke, Australian Institute for Musculoskeletal Science, Melbourne Medical School, Sunshine Hospital, The University of Melbourne, Parkville, VIC, Australia; ^2^School of Psychology and Public Health, College of Science, Health and Engineering, La Trobe University, Melbourne, Parkville, VIC, Australia; ^3^Department of Medicine and Dean's Office, Rajarata University of Sri Lanka, Anuradhapura, Sri Lanka; ^4^Department of Neurology, Australian Institute for Musculoskeletal Science, Level Three, Western Centre for Health Research and Education, Sunshine Hospital, Western Health & University Melbourne, St Albans, VIC, Australia; ^5^Faculty of Social and Political Sciences, Tbilisi State University, Tbilisi, Georgia

**Keywords:** acute ischemic stroke, COVID-19, neurorehabilitation, white blood cells, neutrophil lymphocyte ratio, hyper coagulopathy, D-dimer, ferritin

## Abstract

Corona virus disease (COVID-19), caused by the severe acute respiratory syndrome coronavirus2 (SARS-CoV-2) is recognized as a global pandemic by WHO 2020 with 5,934 936 infections, 367,166 deaths and affecting over 200 countries as of 30th May 2020. Acute Ischemic Stroke (AIS) in brain is also emerging as an important neurovascular/neurological complication of COVID-19, associated with extreme immune responses leading to dysregulated coagulation system and generalized thrombo-embolic status and increased risk of AIS especially among usually less vulnerable younger adults in this cohort. Thus, in early June 2020, we aimed to review the clinical data on all published cases of COVID-19 and concomitant AIS, with a view to understanding the pertinent clinical, laboratory and imaging features. The neutrophil-lymphocyte ratio (NLR) at time of hospital admission for COVID infection correlates positively with the duration of time before onset of clinical features of AIS. Higher NLR, C-Reactive protein, serum ferritin, D-dimer and fibrinogen levels are associated with poor prognosis of AIS in COVID-19 with 75% of patients dying or being severely disabled at present. Currently it is too early to comment on the long-term outcomes for survivors.

## Key Findings

- Acute ischemic stroke is an important, but an under recognized complication of SARS-CoV2 infection, that leaves most recovered patients with significant disabilities as of present stage July 2020 of the pandemic.
- Hypercoagulation markers such as D-dimer are substantially elevated among all patients early in the disease progression.
- Neutrophil to lymphocyte ratio, C-Reactive protein, and Serum Ferritin levels appear to be prognostic markers.
- Patients with higher admission neutrophil-lymphocyte ratios demonstrate a shorter interval between infective symptoms of COVID-19 and the clinical manifestation of Acute Ischemic Stroke.
- Large vessel occlusion is the main etiologic subtype, with only a minority of patients receiving standard of care treatment.
- Seventy five percent of the patients with COVID-19 and Acute Ischemic Stroke died or are still severely disabled.
- The COVID-19 pandemic has created a unique opportunity to advance the whole field of neurorehabilitation based on a better biological and scientific underpinning of precision neurorehabilitation protocols.

## Introduction

In December 2019, a novel corona virus associated with a series of acute, atypical respiratory diseases was first detected in Wuhan China. Since then the virus, now known as SARS-CoV2 (Severe Acute Respiratory Syndrome coronavirus two), has spread to over 200 countries and is now recognized as a major world pandemic (1). As of May 30th 2020, the mortality rate of COVID-19 was reported with the number of confirmed deaths with recorded cases worldwide. Since the pathogenesis of SARS-CoV2 first began to emerge, numerous other clinical system manifestations have been identified.

Neurological manifestations of SARS-CoV 2 infection were first reported in a series of patients in Wuhan, China by Zhou et al. (2). Acute ischemic stroke (AIS) was diagnosed in 5% of the cases (2). However, a much lower rate of only 0.9% imaging confirmed AIS i.e., 32/3,556 total patients case number with COVID-19 was reported in New York USA (3). Subsequent retrospective reports from Europe have also confirmed AIS as a common neurovascular complications of SARS-CoV2 (4, 5). Interestingly Oxley et al. noted an increased occurrence of younger SARS CoV2 virus-infected patients with no significant traditional risk factors for AIS, presenting with large vessel occlusion (6). Putative mechanisms suggested as inducing AIS in association with SARS CoV2 have included systemic inflammation, inflammatory cytokine storm, hyper-coagulability, and imbalances in the classical and alternative Renin Angiotensin System (RAS) in relation to SARS-CoV-2 spike glycoprotein-ACE2 binding related molecular mechanisms (3, 7–19). The RAS system comprises both a plasma-based RAS regulating cardiovascular system and tissue-based RAS regulating long term changes via a complex hormonal system, endocrine, paracrine, and autocrine in action. Thus, the RAS controls renal, adrenal and cardiovascular systems with important implications on blood pressure control as well as fluid/electrolyte control which are critically important to maintain life being very susceptible to damage by SARS-CoV 2. The inflammatory pathway is core to the various clinical manifestations of SARS-CoV2 infection. Also referred to as the “cytokine storm,” it triggers an upsurge of various inflammatory cytokines such as IL-2, IL-7, IL-10 (20, 21), induces a state of lymphocytopenia (22–24) and also activates a spike of acute phase reactants such as CRP and ferritin (25, 26).

Various parameters have been proposed to predict prognosis and outcomes among patients with COVID, including the neutrophil to lymphocyte ratio (NLR) (27–30). A metanalysis of six studies involving 1,141 patients has demonstrated that an elevated NLR is associated with severe disease manifestation (28). The same meta-analysis has also revealed that along with ESR and IL-6, CRP was correlated with increased severity among patients with SARS-CoV2 infection (28). The role of ferritin as a predictor of mortality among confirmed SARS-CoV2, has also been confirmed in another metanalysis of 10 studies involving more than 1,400 subjects (31). Furthermore, elevated D-dimer and hyperfibrinogenemia, which are both biomarkers of inflammation and hypercoagulable state, have also been shown to predict the severity of the said infection (31, 32). Interestingly, similar biomarkers predict outcomes in stroke (33–39). In particular, it is known that patients who show elevated NLR, ferritin, CRP, D-dimer and fibrinogen have a higher risk for stroke and equate to potentially poorer clinical outcomes (33–39).

To date, despite the theoretical association of inflammatory and procoagulable states linking stroke and SARS-CoV2 infection, there is limited published literature on the actual co-occurrence of both. There is also limited information on the biological markers which may be associated with poor neurological outcomes. Thus, this study aims to describe the clinical characteristics of patients with acute ischemic stroke and concomitant SARS-CoV2 infection. By further analysis of available laboratory data, this will look at the trend of inflammatory biomarkers such as NLR, CRP, serum ferritin, fibrinogen and D-dimer and hospital discharge outcomes.

Currently, there is limited information about the clinical characteristics and specific neurorehabilitation issues of AIS patients with SARS-CoV 2 infection (40–43). However, it is expected that the surge in patient numbers, on-going issues with personal protective equipment (PPE) shortages, and associated health care workers anxiety and stress about the potential of getting infected with COVID-19 (and actual infection of health care workers and mandatory self isolation for 14 days even if these members are demonstrating minimum or no symptoms) will create a significant challenge to traditional neurorehabilitation practices and pathways, at least during the pandemic, possibly for a long time to come. Thus, these circumstances argue a strong case for converting the catastrophe [Complex rearrangement of hospital facilities as part of the preparation for the pandemic has also occasioned significant problems and added resource problems for health care systems across the world (44–50) into an opportunity for revamping of rehabilitation protocols]. Currently evidence is emerging for further expansion of telemedicine type paradigms, with incorporation of tablet based remote monitoring technology (Melbourne Rapid Field visual fields, wearable devices and artificial intelligence) suggesting as the way forward in neurorehabilitation of AIS in COVID19 pandemic era, at least for the foreseeable future (43, 51–53).

Thus, this systematic review aims to identify and collate the clinical and laboratory features, acute and long term treatment, and outcomes of all published reports on patients with concomitant diagnosis of confirmed SARS-CoV 2 infection and acute ischemic stroke and with a special emphasis on clinical and laboratory features.

## Purpose

The present study was conducted to provide a systematic review of AIS and COVID-19 with respect to definition, prevalence, pathophysiology, clinical characteristics, acute, subacute features, prognostic markers outcomes.

### Participants

Information regarding ischemic stroke patients with confirmed SARS-CoV2 infection and radiologically or clinically Confirmed AIS included in published studies from November 2019 to May 30th 2020 using the search strategy detailed below will be considered here.

### Types of Studies

All types of studies including qualitative, systematic reviews, meta-analyses, case reports and case series, were included.

### Search Methods

Published articles in English and on human subjects that were published from November 2019 until 30th May 2020 were the inclusion criteria for the search. The following search strategy was adopted:

1. In the first step MEDLINE, Cochrane and CINAHL databases were searched, followed by title and abstract search.
2. In the second step, the keywords were used when searching on Ovid MEDLINE, Cochrane, PubMed, CINAHL, and EMBASE databases.
3. In the third step, a manual search was carried out to ensure no study was inadvertently left out.

The keywords used to conduct the search were: Stroke, thrombosis, coronavirus, neurological complication, neurorehabilitation, COVID19, SARS-COV2.

### Data Extraction

The Arksey and O'Malley methodological framework was employed in this review (54).

The bibliographies of individual studies were further hand-searched. Articles were screened by two independent investigators.

4. In the fourth step the secondary analysis was carried out as follows.

Clinical and laboratory data of every patient was extracted. Demographics and details of their respective laboratory details were also investigated. In particular, the following routine laboratory values were of interest to the researchers: NLR, CRP, ferritin, fibrinogen, and D-dimer. Individual patient outcomes were also accounted for and classified as good [with modified Rankin Scores (mRS) of 0, 1, 2, and 3 and poor mRS of 4, 5, 6]. Patients with no available laboratory data and outcomes were excluded in the quantitative analysis.

## Search Results

Extensive database search yielded 595 citations, and four studies were added by manual searching. A total of 257 duplicates were excluded resulting in 342 citations. These titles and abstracts were further screened yielding 90 final publications of relevance to consideration of stroke and SARS-COV2 infection, during the second screening process. One publication was non-existent despite being cited by multiple authors in their publications. Further evaluation of the full texts of the 89 studies by two independent neurologists (TW and CS) excluded 74 citations with 15 studies. Three further studies were added from hand-held search by TW and CS with 18 publications that were deemed to be included in this systematic review by all authors.

### Year and Country of Study

The studies published from 2019 to 2020, Included literature were originated from North America, Europe, and Asia.

### Study Population

This study included all patients with SARS-COV2 infection and a concomitant diagnosis of acute ischemic stroke and/or acute/subacute outcomes where available.

**Figure 1:**
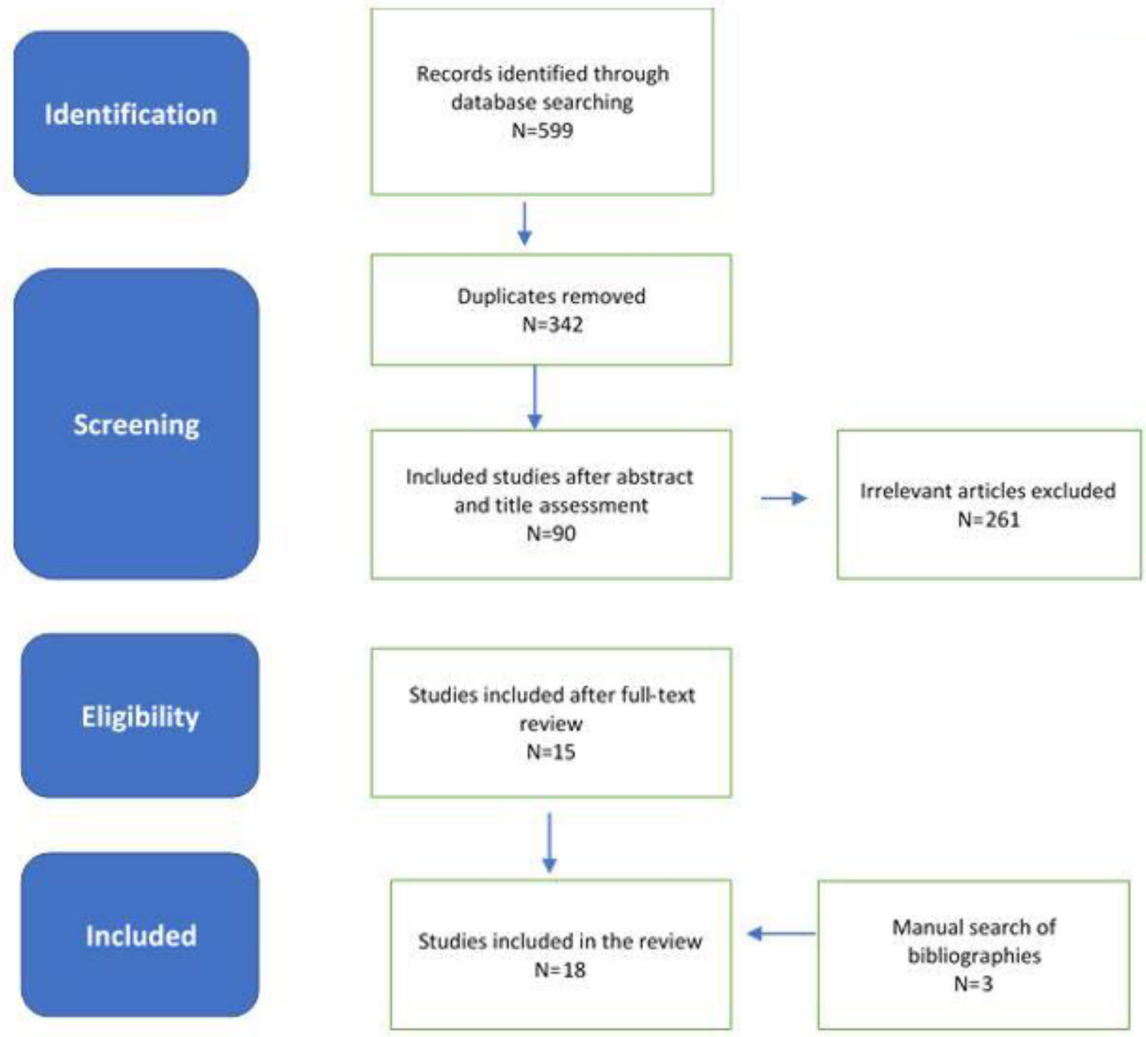
PRISMA Flowchart.

An electronic search performed on May 10 to 30th, 2020 using the identified keywords yielded 342 citations after removal of duplicates. This was further assessed at the title and abstract level which resulted in 90 articles. After full assessment of the full text of each, 18 were deemed relevant to the study, in addition to the three articles which were added from hand-held research. Figure 1 summarizes the search process.

There were 18 articles included in the study consisting of 87 patients from USA, Italy, Turkey, France, Philippines, and United Kingdom. Most of the studies were case reports and case series while three of the included studies were retrospective and prospective cohorts. Table 1 outlines the characteristics of the individual studies.

**Table 1:** Characteristics of studies included.

**Author**	**Country**	**Study design**	**Number of patients with confirmed SARS-CoV2 infection and AIS**
Yaghi et al. (3)	USA	Retrospective cohort	32
Lodigiani et al. (4)	Italy	Prospective cohort	8
Berekashvilli et al. (55)	USA	Prospective cohort	10
Wang et al. (38)	USA	Case series	5
Beyroutti et al. (56)	UK	Case series	6
Avula et al. (57)	USA	Case series	4
Tunc et al. (58)	Turkey	Case series	4
Oxley et al. (6)	USA	Case series	5
Morassi et al. (59)	Italy	Case series	4
Viguier et al. (60)	France	Case report	1
Co et al. (61)	Philippines	Case report	1
Deliwala et al. (62)	USA	Case report	1
Al Saiegh et al. (63)	India	Case report	1
González-Pinto et al. (64)	Spain	Case report	1
Gunasekaran et al. (65)	USA	Case report	1
Valderrama et al. (66)	USA	Case report	1
Moshayedi et al. (67)	United Kingdom	Case report	1
Goldberg et al. (68)	USA	Case report	1

Clinical characteristics of patients are described in Table 2.

**Table 2:** Clinical characteristics of patients described.

Age (years) (*N* = 87)	>70	29 (33%)
	50–70	38 (43%)
	<50	21 (24%)
Comorbidities (*N* = 87)	Hypertension	46 (53%)
	Diabetes	32 (37%)
	Dyslipidemia	21 (24%)
	Atrial Fibrillation	8 (9%)
Time from SARS-CoV2 symptoms to onset of stroke (days) *N* = 60	> 14	19 (31%)
	7–14	17 (28%)
	<7	24 (40%)
Laboratories	Mean hemoglobin (g/L)	129 (94–155)
	Mean white cell count (/mm^3^)	10.23 (0.5–23.05)
	Mean platelet count (/mm^3^)	269.41 (135–408)
	Neutrophil to lymphocyte ratio (NLR)	6.99 (0.91–17.4)
	Mean creatinine (umol/L)	129.81 (55–537)
	Mean fibrinogen (g/L)	5.52 (1–9.7)
	Mean D-dimer (ug/L)	9,800.47 (52–000)
	Mean INR	1.48 (0.99–3.6)
	Mean APTT (s)	31.66 (24–42.7)
	CRP (mg/L)	131.817742 (4–366)
Neuroimaging *N* = 35	Any stenosis	5 (13%)
	Middle cerebral artery occlusion	15 (43%)
	ICA/CCA occlusion	8 (23%)
	Anterior cerebral artery occlusion	1 (3%)
	Tandem occlusion	2 (6%)
	Posterior cerebral artery occlusion	2 (6%)
	Basilar artery occlusion	1 (3%)
	Posterior inferior cerebellar artery occlusion	1 (3%)
Treatment *N* = 69	Alteplase only	4 (6%)
	Thrombectomy	3 (4%)
	Alteplase and thrombectomy	8 (12%)
	Alteplase and thrombectomy and antiplatelet/anticoagulation	5 (7%)
	Thrombectomy and antiplatelet	4 (6%)
	Anticoagulation only	32 (46%)
	Antiplatelet only	8 (12%)
	Anticoagulation and antiplatelet	5 (7%)
Outcomes *N* = 72	MRS 3 and below	17 (24%
	MRS 4 and above	55 (76%)

The majority of the patients were within the 50–70 age group while almost one-third of the patients were <50 years old. The most common comorbidity was hypertension followed by diabetes, dyslipidemia and less frequently, atrial fibrillation. Mean hematologic parameters are also described. Neurovascular imaging either with magnetic resonance angiography (MRA) or computer tomographic angiography (CTA) was available for 35 patients, of whom the majority presented with anterior circulation, large vessel occlusion. Treatment regimens were also described for the majority of the patients and among whom a significant number received systemic anticoagulation, intravenous thrombolysis and mechanical thrombectomy. Of the 87 patients described, 72 outcomes are available, with almost 75% resulting in poor neurological outcomes of Modified Rankin score (mRS) 4 and above.

**Figure 2:**
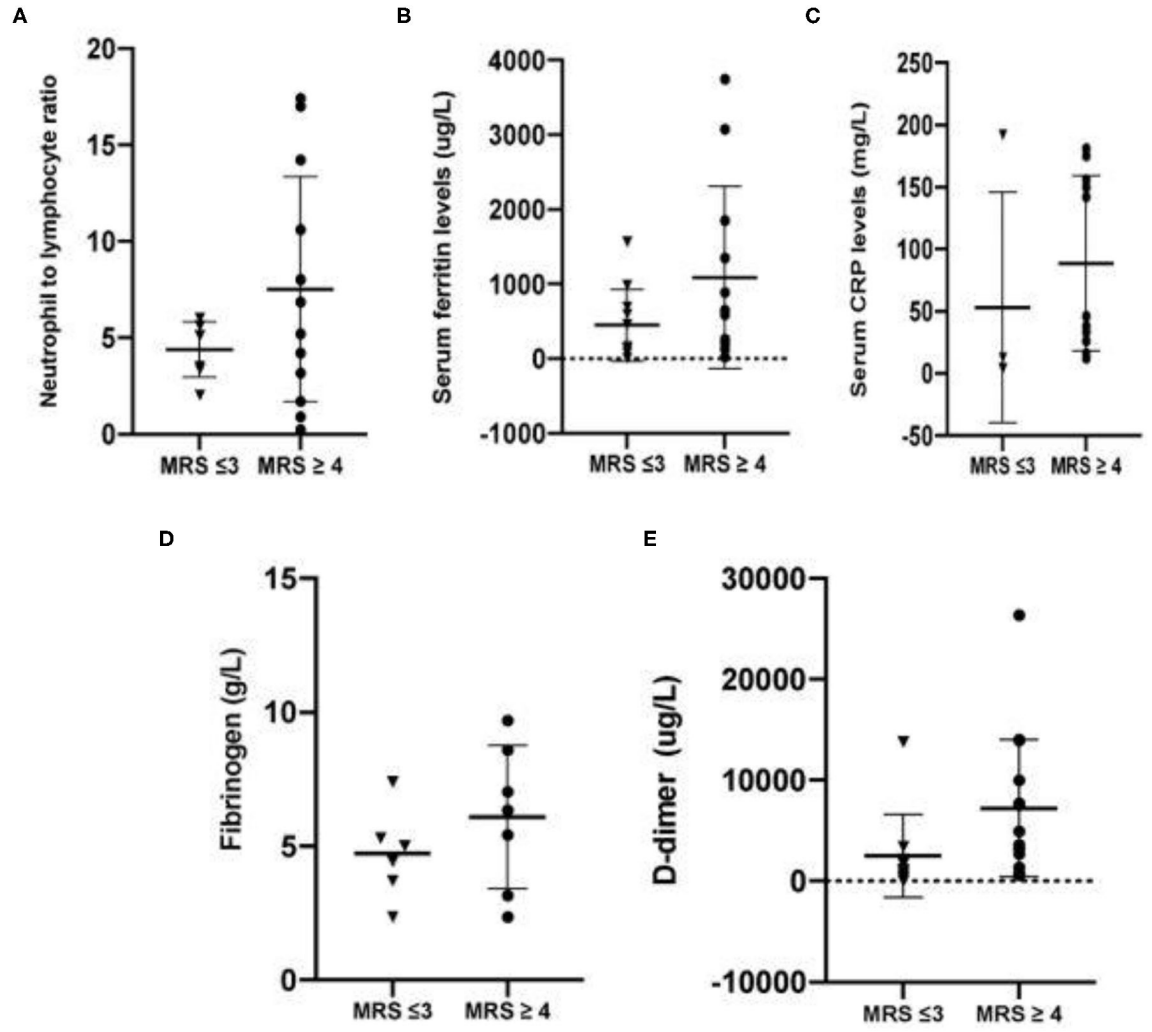
**(A–C)** Mean inflammatory markers among patients with stroke and confirmed SARA-CoV2 infections. **(D–E)** Mean coagulation markers among patients with stroke and confirmed SARS-CoV2 infection.

Inflammatory and coagulation markers of individual patients were also analyzed. Neurological outcomes were classified as either good (mRS 3 and below) or poor (mRS 4 and above). Respective inflammatory parameters such as neutrophil to lymphocyte ratio, C-reactive protein and serum ferritin were analyzed for each group. The same was performed for coagulation markers such as D-dimer and fibrinogen. Patients with good neurological outcomes had lower mean NLR, CRP and serum ferritin (4.39 ± 1.44, 53.09 ± 92.70 mg/L, 449 ± 482.3 ug/L, respectively), compared to patients with mRS 4 and above (7.51 ± 5.84, 88.69 ± 70.45 mg/L, 1,086 ± 1,220 ug/L, respectively). Similar trends were observed in terms of coagulation markers, with D-dimer and fibrinogen showing levels of 2,509 ± 4,093 ug/L and 4.70 ± 1.70 g/L, respectively, for patients with mRS 3 and below, while values for patients with poor neurological outcomes were 7,223 ± 6,781 ug/L for D-dimer and 6.086 ± 2.69 g/L for fibrinogen respectively. Summary of the said values are plotted in Figures 2A–E.

**Figure 3:**
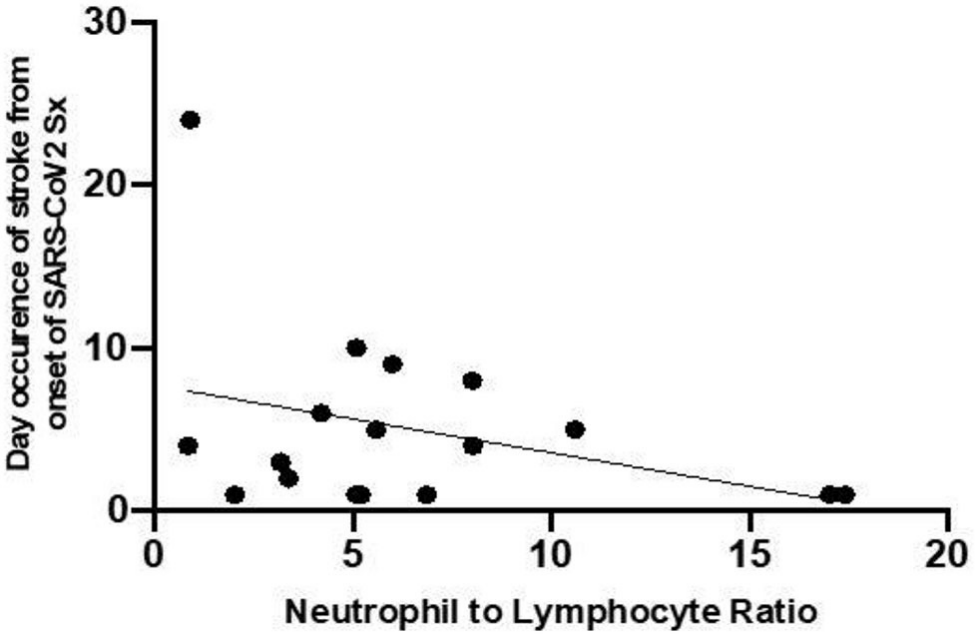
Relationship between NLR and the occurrence of stroke from onset of SARS-CoV2 symptoms.

The relationship between the NLR on admission and the time interval from onset of SARS-CoV2 symptoms to the appearance onset of symptoms of stroke was established. As shown in Figure 3, patients who have higher NLR at the onset have a shorter time interval between infective symptoms and the occurrence of the ischemic event.

## Discussion

To date, there is no comprehensive review describing the potential role of inflammatory and coagulation biomarkers in determining the clinical outcomes of patients with SARS-CoV2 infection and concomitant acute ischemic stroke. The data presented will also supplement currently limited information on the occurrence of neurovascular events among patients with SARS-CoV2 infection.

To date a number of theoretical models have been proposed to account for the occurrence of neurovascular events among SARS-CoV2 patients. Most build on the idea of the SARS-CoV2 virus infection inducing inflammation and associated immunological release of cytokines from blood and endothelial cells and the concurrent activation of platelets resulting in micro thrombosis (69). The depletion of the cardioprotective and neuroprotective ACE-2 receptors throughout the body and on microglia in the brain, as a result of the receptors being the preferential cellular target of the virus invasion, has also been proposed as another neuropathologic mechanism irrespective of age (8). However, the hypercoagulable state of SARS-COV2 infection as the sole basis of this mechanism is debateable given that vascular workups for cryptogenic stroke have not been detailed in most of the case studies. Furthermore, the increase in “burden of disease” especially in the elderly is likely to be further exacerbated by the expected age-related depletion in ACE-2 receptors resulting in the predominance of the end-organ damaging effects of increasing the ACE-1/Angiotensin II ratio (70–72).

To date, the majority of AIS lesion sites in the patients described in the literature, are related to large vessel occlusion. However, it remains unclear whether this is due to a mechanism related to thrombosis or embolism or the lack of brain imaging. Unfortunately, there are no studies to date, which fully report autopsy findings of the deaths recorded among the stroke patients with SARS-CoV infection. In a different, though recent, study describing the autopsy results of 12 SARS-CoV2 patients in a German center, the majority of cases showed massive venous thromboembolism with no arterial thrombosis being reported (73). Mechanisms which may contribute to intracranial arterial thrombosis include the cytokine-induced initiation of thrombin formation that triggers the activation of platelets that subsequently result in the development of micro and macrothrombi (74–87). This is worsened by the free conversion of fibrinogen to fibrin and inflammation-induced depletion of physiological anticoagulants such as antithrombin III, tissue factor pathway inhibitor, and the protein C system (74–88). In terms of treatment, while 30 cases were reported to have large vessel occlusion, only 20 mechanical thrombectomies were performed. A comprehensive stroke center in Barcelona, Spain reported an 18 and 23% drop in the number of strokes codes and mechanical thrombectomies during the start of the pandemic, respectively, albeit without any changes in reperfusion and clinical outcomes (89) The World Stroke Organization recognizes the said difficulties and emphasizes the utility of telemedicine as well as best practice sharing to further optimize and streamline stroke processes (90, 91).

While not depictive of the true epidemiologic picture, it is clear that patients with AIS and SARS-CoV2 infection have poor neurologic outcomes of either death or severe disability. Aggarwal et al. (92) concluded in a point analysis of four studies that patients with a previous history of stroke have a 2.5-fold increase in the odds of severe COVID infection but did not show any significant association with mortality (92). A retrospective cohort study of ischemic stroke reports a mortality rate close to 50% (3) while a prospective study involving 10 AIS patients resulted in four deaths (55) Clearly, more prospective studies involving a larger number of individual patients is necessary to ascertain the true mortality rate in this population.

In this study, there is a trend that patients with good outcomes have lower NLR, CRP, and serum ferritin compared to patients who died or remained critically ill. NLR has been shown to have a good predictive value in assessing patients who are likely to have severe SARS-CoV2 infection (30, 93–96). In particular, it has been proposed that patients who are older and have NLR values of more than 3 are likely to require intensive care (27). Yan et al. also predicted that high NLR values on admission is associated with greater odds of complications related to COVID-infection (97). On the other hand, it is known that high NLR is used as a poor prognosticating factor for patients with cerebral ischemia, intracerebral hemorrhage and post-stroke complications (98–107). The dual consequence of COVID-related lymphopenia along with migration of the neutrophils to the ischemic tissue may contribute to the significant increase in the NLR levels in patients with stroke and concomitant SARS-CoV2 infection (107).

Another hyperinflammatory biomarker which has been shown to stratify outcomes in patients with SARS-CoV2 infection is CRP. Aside from predicting severity and mortality, it has prognosticating value in determining which patients will eventually require mechanical ventilation (108–110). Published literature noted that elevated CRP is associated with poor outcomes in patients with neurovascular conditions (111, 112). There is also evidence to suggest that CRP is not just a “marker” but a “maker” of the atherogenesis (110). It has been demonstrated in experimental studies that exogenous CRP promotes atherogenesis by promoting the expression of adhesion molecules and cell mediators along with the decrease of arterial vasodilators (113–115). A meta-analysis of nine studies also provides evidence on the dose-dependent relationship of CRP and increased risk of venous thromboembolism (112). Whether the elevation of CRP is the causative etiology or the sequelae of a multifactorial process linking SARS-CoV2-infection to inflammation, atherogenesis or embolism needs further exploration.

Hyperferritinemia, which implies a heightened state of immunologic reactivity has also been associated with increased mortality in recent publications related to the SARS-CoV2 infection (116). It signals the activation of the macrophages and the reticuloendothelial system resulting in end-organ damage (117). Patients with SARS-CoV2 treated for pneumonia with Toculizumab had a marked decrease in the inflammatory markers such as CRP and ferritin, along with significant clinical improvement post-infusion (118). In patients with acute stroke, this iron storage protein can potentially worsen the iron-dependent oxidative stress in the ischemic penumbra which can lead to further neurologic decline (119). This is further validated in a study which shows a direct correlation between serum ferritin and markers of neural and blood-brain barrier disruption such as glutamate, interleukin-6, matrix metalloproteinase-9 and cellular fibronectin among patients receiving thrombolysis (39). The complementary inflammatory sequelae of SARS-CoV2 infection and ischemic stroke is the likely culprit of hyperferritinemia in SARS-CoV2 related strokes.

SARS-CoV2-related coagulopathy is responsible for various thrombotic events linked to mortality. Described as a fibrinolytic “shut-down,” SARS-CoV2 infection promotes a pro and hypercoagulable states resulting in disseminated (intravascular coagulation (DIC), microthrombi and other venous and arterial thrombotic phenomena (4, 120–122). D-dimer and fibrinogen are both recognized as important biomarkers of the severity of coagulopathy in patients with SARS-CoV 2 infection (123, 124). Olive et al. in a retrospective analysis of 21 patients with SARS-CoV infection concludes that D-dimer was associated with increased risk of pulmonary embolism (125). A similar observation was made in a larger study that suggests that D-dimer levels above 1 μg/mL may help in stratifying patients with poor prognosis at the onset (26). Fibrinogen increase was also observed among patients with severe SARS-CoV2 related pneumonia compared to mild presentation (126). The disproportionate increase of these biomarkers, especially at the early stages, warrant screening of thromboembolic events and initiation of thromboprophylaxis (124). The trend in these coagulation biomarkers are similarly observed in non-COVID related strokes. In the ARISTOTLE trial, patients with AF and increased D-dimer values had higher incidence of stroke, systemic embolism and all-cause mortality (127). Choi and colleagues also propose that D-dimer can be used as a biomarker for recurrence among patients with previous AF and non-AF related strokes (128). The EUROSTROKE study likewise confirms the utility of fibrinogen in predicting patients who are at risk for stroke (36). The said risk is equated to various clinical risks such as smoking, DM, MI, and HDL cholesterol (36). In this study, we have provided a scaffold on the potential trend between outcomes and coagulation parameters for SARS-CoV2 related strokes. While the most accepted mechanism behind this phenomenon is sepsis-induced disruption of the coagulation system, Iba et al. propose that more complex procoagulant responses resulting in a distinct interaction between the host's immunologic and the coagulation systems (124).

This study also highlights the occurrence of the ischemic event days to weeks after the onset of SARS-CoV2 symptoms. More importantly, we have established an inverse relationship between the inflammatory biomarker, NLR on admission and the duration between the stroke and the onset of SARS-CoV2 symptoms. This is likely related to the inflammatory burden which triggers a pro-coagulable cascade. Furthermore, Amiral et al. relate this to the alloimmune hypothesis, which has been demonstrated in rodents (129). The development of auto-antibodies to other ACE-2 receptors such as on the microglia in the brain after the onset of viral infection presumably resulted in the exponential increase in the cytokine storm and significant tissue destruction which may be linked to the delayed onset of the vascular event after the viral prodrome (129).

Lastly as the COVID-19 pandemic is distressing national health systems worldwide, a tsunami wave of neurorehabilitation needs and challenges regarding the long-term effects of the pandemic must be expected to begin to unfold soon. Thus, we believe that with strong humanity and collaboration across disciplines, this is the time to convert this situation into an opportunity that with vision, creativity, innovation, and use of smart technology can be harnessed with the aim of surviving this global health crisis (43, 130).

## Conclusion

Stroke is an important neurovascular complication of SARS-CoV2 infection. The aetiopathogenesis of cerebral ischemia is related to the overactivation of immune and hypercoagulable mechanisms. This is supported by the disproportionate increase of biomarkers such as NLR, CRP, serum ferritin, D-dimer and fibrinogen among patients who died or were critically ill. An elevated NLR on admission also implies an increased burden of inflammation at the onset of SARS-CoV infection which may result in early manifestation of cerebral ischemic events.

## Author Contributions

TW and LK conceived of the presented idea. TW, SC, and LK developed the theory. TW and CS performed the literature search. TW wrote the manuscript with support from CS, LK, and SC. All four authors approved the final manuscript.

## Conflict of Interest

The authors declare that the research was conducted in the absence of any commercial or financial relationships that could be construed as a potential conflict of interest.
